# Comparative Safety and Efficacy of Left Atrial Appendage Occlusion Technologies for Prevention of Stroke and Bleeding in Atrial Fibrillation: A Comprehensive Meta-Analysis of Watchman Versus Oral Anticoagulation and Alternative Device Platforms

**DOI:** 10.7759/cureus.100284

**Published:** 2025-12-28

**Authors:** Efe Opone, Nafees Khan, Sidra Jabeen

**Affiliations:** 1 Internal Medicine, University Hospitals Sussex, Brighton &amp; Hove, GBR; 2 Medicine and Surgery, Liaquat National Hospital and Medical College, Karachi, PAK

**Keywords:** anticoagulation, atrial fibrillation, bleeding, left atrial appendage occlusion, meta-analysis, mortality, stroke, watchman device

## Abstract

Left atrial appendage occlusion (LAAO) offers an alternative to oral anticoagulation for stroke prevention in atrial fibrillation. We performed a systematic review and meta-analysis comparing Watchman (Boston Scientific Corporation, Marlborough, MA) with oral anticoagulants (OACs) and alternative LAAO devices. PubMed, EBSCO, and ClinicalTrials.gov were searched (January 2005 to August 2025) for randomised trials and observational studies comparing Watchman with OACs or other devices in adults with atrial fibrillation (minimum one-month follow-up). Twenty-one studies (eight randomised, 13 observational; n=12,357) were analysed. Results reflect combined randomised and observational evidence, with heterogeneity addressed using random-effects models. Most comparators were warfarin; DOAC-specific data were limited. Versus OACs, LAAO reduced transient ischaemic attack (odds ratio (OR) 0.72), haemorrhagic stroke (OR 0.41), all-cause mortality (OR 0.73), and cardiovascular mortality (OR 0.57), with similar ischaemic stroke rates (OR 1.09). Ten studies contributed device-device comparisons. Versus other devices, Watchman had fewer complications (OR 0.67) but more major peridevice leaks (OR 1.84). Watchman showed a consistently more favourable safety profile compared to others. Device-related complications were significantly lower (OR 0.67), including markedly reduced device embolisation (OR 0.45) and procedural bleeding (OR 0.68). Watchman also demonstrated fewer major procedural complications and a lower rate of pericardial effusion requiring intervention. Efficacy outcomes, including ischaemic stroke, transient ischaemic attack (TIA), haemorrhagic stroke, and systemic embolism, were comparable across devices, with no significant differences detected. Follow-up duration varied across studies, though absolute event rates remained low. Publication bias and residual confounding may influence mortality estimates, as device-generation reporting was incomplete. In conclusion, LAAO reduces haemorrhagic stroke and mortality compared with OACs while maintaining equivalent ischaemic stroke protection. Among devices, Watchman demonstrates superior procedural safety but increased leak rates.

## Introduction and background

Atrial fibrillation substantially increases the risk of stroke and systemic thromboembolism by promoting atrial stasis and facilitating thrombus formation within the left atrial appendage, the source of most AF-related emboli [1]. The global healthcare burden of atrial fibrillation continues to expand at an unprecedented rate. Contemporary epidemiological data reveal that the worldwide prevalence has reached 59.7 million individuals as of 2019, representing a dramatic 78% surge since 2010 [2]. Within the United States alone, a comprehensive analysis of electronic health records encompassing 259 million patients identified 4.8 million individuals with atrial fibrillation, demonstrating a concerning upward trajectory in prevalence from 4.49% to 6.82% over just four years [3]. Untapped and unregistered data from lower- and middle-income countries may reveal an even more uncontrolled trajectory of these trends. The rising global burden of atrial fibrillation, increased uptake of direct oral anticoagulants (DOACs), and expanding needs for stroke prevention directly heighten the clinical importance of determining whether left atrial appendage occlusion (LAAO) provides a net benefit over oral anticoagulation. Additionally, there is a critical need to identify which LAAO device best suits specific patient profiles, particularly among individuals with contraindications to anticoagulation, limited medication adherence, or complex bleeding risks.

Contemporary management guidelines have undergone significant evolution in their approach to thromboembolic risk stratification and prevention. Guidelines have evolved in their approach to thromboembolic risk stratification and prevention. Both American and European cardiology societies emphasise individualised risk assessment based on annual stroke probability rather than rigid scoring thresholds while establishing DOACs as the preferred anticoagulant choice over vitamin K antagonists (VAKs) [4]. Comprehensive patient-level meta-analyses encompassing 1,312,609 participants have validated this preference, demonstrating that DOACs significantly reduce risk by approximately 41% versus warfarin [5].

Nevertheless, substantial challenges persist in implementing effective anticoagulation strategies across real-world patient populations with atrial fibrillation. Medication persistence remains suboptimal, with longitudinal cohort studies documenting that fewer than half of patients maintain consistent DOAC therapy at 12 months, while warfarin adherence is approximately 40% [6]. This suboptimal adherence correlates with increased rates of both thromboembolic events and mortality. Additionally, a considerable subset of patients harbour absolute or relative contraindications to systemic anticoagulation, including those with histories of life-threatening haemorrhage, recurrent bleeding despite optimal management, or conditions predisposing to traumatic injury [3].

Recognition of the left atrial appendage as the principal origin of cardioembolic thrombus in non-valvular atrial fibrillation has catalysed the development of mechanical exclusion techniques. Pathological and imaging studies demonstrate that this trabeculated cardiac structure harbours approximately 90% of atrial thrombi in patients with non-rheumatic atrial fibrillation [7]. This anatomical insight provided the rationale for developing percutaneous closure devices capable of isolating the appendage from systemic circulation.

The clinical validation of percutaneous LAAO emerged through pivotal randomised trials that established both safety and efficacy benchmarks. The PROTECT AF trial investigated 707 patients randomised between Watchman implantation (Boston Scientific Corporation, Marlborough, MA) and warfarin therapy, ultimately demonstrating a 40% relative risk reduction in the primary composite endpoint, accompanied by substantial reductions in both haemorrhagic stroke (85% decrease) and mortality (34% decrease) [8]. Building upon these findings, the PREVAIL study incorporated refined procedural techniques and operator training requirements, successfully achieving non-inferiority while documenting marked improvements in procedural safety, with complication rates declining from 8.7% to 2.2% [9]. It is important to note that these pivotal trials primarily compared LAAO to warfarin rather than contemporary DOACs, potentially limiting their applicability to current practice. Furthermore, patient selection in these trials may not fully represent the broader atrial fibrillation population, as participants were carefully selected based on specific inclusion criteria. In addition, device-device and device-generation differences influence sealing, anchoring, and complication profiles, making generation-level distinctions critical to interpretation, which have not yet been addressed in a single trial.

Technological advancement has yielded multiple device iterations and novel design approaches aimed at optimising procedural success and minimising complications. The Watchman FLX system incorporates design modifications, including additional anchoring barbs and increased fabric coverage, achieving procedural success rates exceeding 98%, with major complications occurring in fewer than 1.4% of cases based on national registry data [10]. Alternative device architectures have also emerged, most notably the Amplatzer Amulet platform, which employs a distinctive dual-component design intended to enhance conformability across diverse appendage morphologies [11].

Contemporary investigations have extended beyond comparisons with warfarin to evaluate LAAO performance against modern anticoagulation regimens. The PRAGUE-17 study specifically enrolled patients at elevated bleeding risk, randomising participants between appendage closure and DOAC therapy [12]. Multiple large-scale trials currently underway, including CHAMPION-AF (NCT04394546) and CATALYST (NCT04226547), will provide definitive evidence regarding LAAO positioning relative to DOACs across broader patient populations. There is still a significant requirement for inter-device interpretation for individual patient-tailored treatment strategies.

Knowledge gaps persist despite accumulating supportive evidence, particularly regarding optimal patient selection algorithms, comparative device performance, and long-term durability. The present systematic review and meta-analysis synthesises available randomised and observational data to provide updated estimates of LAAO effectiveness relative to medical therapy while examining differential outcomes between contemporary device platforms, with particular focus on elucidating safety and efficacy distinctions between market-leading systems.

## Review

Methods

This systematic review and meta-analysis was conducted in accordance with the Preferred Reporting Items for Systematic Reviews and Meta-Analyses (PRISMA) 2020 guidelines [13].

Eligibility Criteria

Studies were eligible for inclusion if they met predefined criteria encompassing the study population, intervention, comparators, outcomes, design, and language. Eligible populations included adults aged 18 years or older with documented atrial fibrillation. The primary intervention of interest was LAAO performed using the Watchman device, including both the first-generation and FLX iterations.

Comparator groups comprised either patients treated with oral anticoagulants (OACs) such as DOACs or VKAs, or those who underwent LAAO with other devices, including but not limited to Amulet, Amplatzer, Plaato, Wavecrest, Occlutech, LAmbre, Ultraseal, PFM, Lariat, and Sierra systems.

Studies were required to report at least one clinically relevant outcome, which could include stroke or systemic embolism, transient ischaemic attack (TIA), major bleeding, all-cause mortality, cardiovascular mortality, myocardial infarction, or any device- or procedure-related complication. Other outcomes of interest encompassed net adverse clinical events (NACE), pericardial effusion, device embolisation, device thrombosis, major vascular complications, procedural bleeding, procedural stroke, and peri-device leak.

Eligible study designs included randomised controlled trials (RCTs) as well as prospective or retrospective cohort studies and registry-based analyses, provided they had a minimum follow-up duration of one month. Finally, only full-text articles published in English were considered for inclusion in this review.

Case reports, case series, reviews, editorials, conference abstracts without full data, and studies without extractable numerical outcomes were excluded.

Search Strategy

A comprehensive systematic search was conducted across three electronic databases (PubMed, EBSCO, and ClinicalTrials.gov) from January 1, 2005, to August 25, 2025. Google Scholar was referred to for grey literature. The search strategy combined controlled vocabulary terms (Medical Subject Headings (MeSH)) with free-text keywords, including the following and using them respectively in each database: "(left atrial appendage closure OR occlusion OR Watchman OR Watchman FLX OR Plaato OR Wavecrest OR Occlutech OR LAmbre OR Ultraseal OR Pfm OR Lariat OR Sierra OR AMPLATZER OR Amulet) AND Atrial Fibrillation." Reference lists of included studies and relevant systematic reviews were manually screened to identify additional eligible publications.

Study Selection

**Figure 1:**
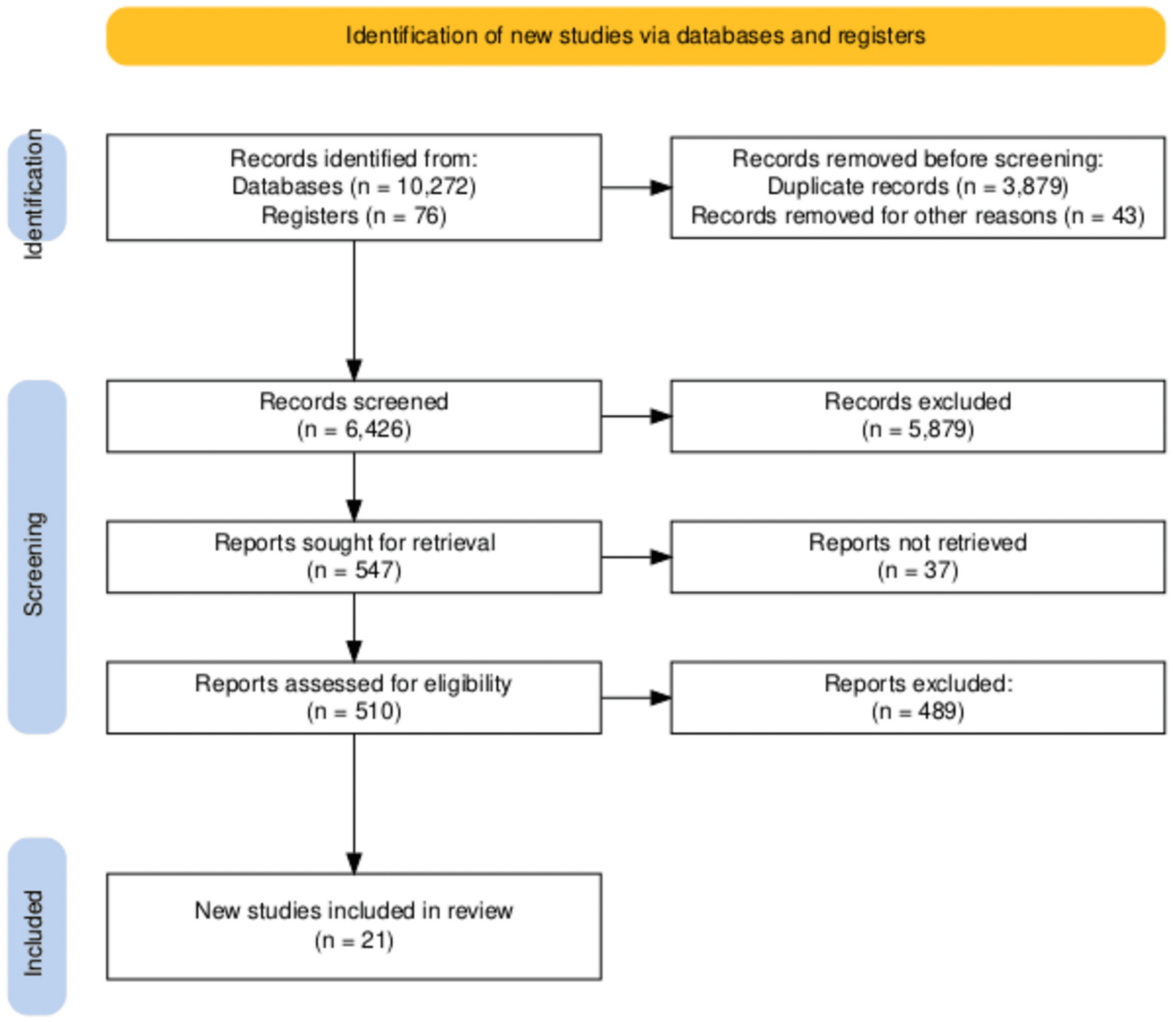
PRISMA 2020 flow diagram: Database searches identified 10,348 records. After removing duplicates and screening, 21 studies met inclusion criteria. PRISMA: Preferred Reporting Items for Systematic Reviews and Meta-Analyses

Two independent reviewers (N.K. and E.O.) screened all retrieved titles and abstracts using predefined eligibility criteria. Full-text articles of potentially eligible studies were independently assessed using a standardised screening form. Disagreements were resolved through discussion, with arbitration by a third reviewer (S. J.) when consensus could not be reached. Reasons for exclusion at each stage were documented and are summarised in the PRISMA flow diagram (Figure 1) [14].

Data Extraction

Data extraction was performed independently by two reviewers using a standardised, piloted extraction form. Extracted variables included study characteristics (first author, publication year, country, study design, sample size, follow-up duration), patient demographics (age, sex distribution, CHA₂DS₂-VASc score, HAS-BLED score, relevant comorbidities), procedural details (device type, anticoagulant regimen, periprocedural antithrombotic protocol), and clinical outcomes at specified time points. When multiple publications reported outcomes from the same cohort, data from the most recent or complete report were used.

Endpoint Definitions

Clinical endpoints were defined according to established criteria (Table 1).

**Table 1:** Clinical endpoint definitions

Endpoint	Definition
Ischaemic stroke	A sudden focal neurological deficit of presumed vascular origin persisting for ≥24 hours, confirmed by computed tomography (CT) or magnetic resonance imaging (MRI), with no evidence of haemorrhage
Haemorrhagic stroke	Symptomatic intracerebral, subarachnoid, or subdural haemorrhage verified by neuroimaging
Systemic embolism	Acute vascular occlusion of an extremity or organ, documented by imaging, surgical exploration, or autopsy, and excluding cerebrovascular events
Major bleeding	Defined according to the International Society on Thrombosis and Haemostasis (ISTH) criteria: fatal bleeding, symptomatic bleeding in a critical organ, or bleeding resulting in a haemoglobin decrease ≥2 g/dL or requiring transfusion of ≥2 units of packed red blood cells
Device-related complications	Composite endpoint including pericardial effusion requiring intervention, device embolisation, device-related thrombosis confirmed by imaging, or major vascular complications necessitating intervention
Peridevice leak	Residual flow around the device detected by transoesophageal echocardiography (TEE) or cardiac computed tomography (cardiac CT), categorised as minor (<5 mm) or major (>5 mm). The measurements were adjusted to our definition of major and minor leaks, where values reported by the studies varied from our definition.

Comparator Devices Details

The devices evaluated in this meta-analysis represent distinct engineering approaches to appendage occlusion. The first-generation Watchman device (Watchman 2.5) employs a self-expanding nitinol frame with 10 fixation anchors and a permeable polyester fabric covering on the atrial-facing surface. Available in five sizes (21-33 mm), it requires a compression ratio of 10% to 20% for optimal stability and seal [15].

The Watchman FLX, in the market since late 2019, represents an evolutionary refinement, featuring 18 fixation anchors (compared to 10 in Watchman 2.5) arranged in two rows, a closed distal end to facilitate atraumatic advancement, and reduced metal exposure on the atrial surface. These modifications allow deployment in appendages with ostial diameters of 14 to 31.5 mm while maintaining 10% to 27% compression [16].

The Amplatzer Amulet (Abbott) utilises a fundamentally different dual-structure design consisting of a distal lobe connected to a proximal disc via a central waist. This nitinol mesh construction, covered with polyester patches, provides immediate ostial coverage through the disc component while the lobe anchors within the appendage body. The device accommodates landing zones from 11 to 31 mm with eight size options [17].

Its predecessor, the Amplatzer Cardiac Plug (ACP), established the concept of separate anchoring and occlusion components but with a smaller disc diameter relative to the lobe and fewer stabilising wires (6-10 versus 10-12 in Amulet). The ACP, available in eight sizes for ostia measuring 12.6-28.5 mm, served as the foundation for understanding dual-component device behaviour [11].

The LAmbre device (Lifetech Scientific) introduces a unique umbrella-and-cover design with multiple small hooks on the umbrella portion for anchoring and a larger fabric-covered disc for ostial sealing. This configuration theoretically allows implantation in challenging anatomies, including shallow or multilobed appendages, with sizes ranging from 16 to 36 mm for landing zones of 12 to 32 mm. Limited availability outside specific markets has resulted in fewer comparative studies [18].

Because the literature disproportionately reports outcomes for the Watchman device, it contributes the largest comparative dataset relative to all other platforms. However, the ACP and Amulet devices remain important comparators in this review, with substantial outcome analyses available, whereas LAmbre was the least frequently used device in the reported literature.

Most included studies followed standard post-procedural antithrombotic protocols, typically involving short-term anticoagulation or dual antiplatelet therapy during endothelialisation, followed by long-term single antiplatelet therapy once device sealing was confirmed.

Risk of Bias and Quality Assessment

Risk of bias was assessed using the Cochrane Risk of Bias 2 (RoB 2) tool [19] for RCTs, evaluating five domains: randomisation process, deviations from intended interventions, missing outcome data, measurement of outcomes, and selection of reported results. Observational studies were evaluated using the Newcastle-Ottawa Scale (NOS) [20], assessing selection of study groups, comparability, and outcome ascertainment. Quality assessment was performed independently by two reviewers, with discrepancies resolved through consensus. PREVAIL [9], PROTECT AF [8], OPTION [21], Amulet IDE [22], and WATCH-TAVR [23] were industry-sponsored with frequent financial ties and funding among investigators. Although adjudication and monitoring were independent, sponsor involvement in study design and analysis posed a high risk. PRAGUE-17 [12] and SWISS-APERO [24], which benefitted from partial public or academic sponsorship, showed comparatively lower concerns, while Fan et al. (2025) [25] faced residual detection bias due to unblinded imaging outcomes. Gonzalez et al. (2014) [26], Fastner et al. (2018) [27], Chen et al. (2019) [28], Cheung et al. (2019) [29], Chiu et al. (2022) [29], and Kretzler et al. (2022) [30] did not receive any financial funding and were completely independent studies. However, Mansour et al. (2021) [31], Kleinecke et al. (2019) [32], Figini et al. (2016) [33], Kim et al. (2016) [34], and Saad et al. (2021) [35] declared financial interests of investigators in the form of consultant fees from the LAAO device manufacturers.

Statistical Analysis

Meta-analyses were conducted using Review Manager (RevMan) version 5.4. For dichotomous outcomes, pooled odds ratios (ORs) with 95% confidence intervals (CIs) were calculated using the Mantel-Haenszel (M-H) method. All results are reported as point estimates with 95% CIs, followed by exact p-values with a minimum of two significant digits. Continuous variables were analysed using inverse variance weighting to calculate weighted mean differences. When studies reported medians with interquartile ranges (IQRs), these were converted to means and standard deviations (SDs). There were no data that needed conversion from hazard ratios (HRs) to ORs.

Given anticipated clinical and methodological heterogeneity, random-effects models (DerSimonian-Laird method) were used for all primary analyses due to expected heterogeneity. Statistical heterogeneity was assessed using the I² statistic, with values of 25%, 50%, and 75% indicating low, moderate, and high heterogeneity, respectively. The chi-square (χ²) test was used to assess heterogeneity, with P<0.10 considered significant.

Prespecified subgroup analyses were conducted based on comparator type (Watchman versus oral anticoagulants, Watchman versus Amulet, Watchman versus ACP, Watchman versus Amulet or ACP (nonspecified), Watchman versus all other LAAO devices. It is important to note that device platforms differ substantially in design, which may influence leak and safety outcomes. Subgroup analyses were attempted where reported, although limited device-generation reporting constrained formal stratification between Watchman 2.5 and FLX. Bleeding definitions were harmonised to the International Society on Thrombosis and Haemostasis (ISTH) criteria where possible. Peridevice leak definitions were standardised into major (≥5 mm) vs minor categories, even when studies had differing categorisation, acknowledging variations in imaging modality and timing. Device-generation stratification (Watchman 2.5 vs FLX) was limited by incomplete reporting but is acknowledged as a key interpretive limitation. Comparator devices (Amulet, ACP, LAmbre) were reviewed narratively. Procedural era and operator experience influenced complication rates in early cohorts. Imaging surveillance varied across studies, affecting follow-up detection of leaks and embolisation. However, the reported data were reliable in the periprocedural window. Sensitivity analyses excluded studies with a high risk of bias and examined the influence of individual studies through leave-one-out analysis, and the results are reported where results were significant. 

Publication bias was assessed using funnel plots and Egger's regression test when ≥10 studies were available for an outcome. Missing baseline characteristics were retained as ‘not reported’ and incorporated into risk-of-bias interpretation. Registry-based studies were cross-checked for overlapping enrolment windows, centres, and authorship to avoid double-counting. There is some possibility of population overlap, however, little, which cannot be effectively traced.

We deliberately included studies with varying follow-up durations (one to 60 months) to enhance real-world applicability. LAAO outcomes occur across varied timeframes-procedural complications manifest within days, device-related thrombosis within months, and long-term stroke prevention over years. Some studies reported median follow-up and also maximum follow-up for primary endpoints. By including this temporal heterogeneity, our analysis captures the full spectrum of clinical outcomes. Restricting to uniform follow-up periods would have excluded important early studies (PROTECT AF [8], PREVAIL [9]) and recent real-world registries, creating selection bias. We acknowledge that long-term endpoints require extended follow-up. Periprocedural and post-implant antithrombotic regimens were extracted; due to marked variability, these differences were incorporated qualitatively into the interpretation of bleeding and thrombotic outcomes. The relatively low event rates for certain outcomes necessitated broader inclusion to achieve adequate statistical power for meaningful comparisons.

Results

Study Selection and Characteristics

The comprehensive literature search yielded 10,348 records (PubMed: 5,605; ClinicalTrials.gov: 76; EBSCO: 4,667). After removing duplicates, 6,436 titles or abstracts were screened. Of these, 546 full-text articles were reviewed, and 21 studies met the inclusion criteria. Reasons for exclusion at the full-text stage included either insufficient follow-up duration, lack of a comparator group, duplicate patient populations, insufficient outcome data, wrong intervention, and conference abstracts only. The PRISMA flow diagram summarising the selection process is provided in Figure 1.

The meta-analysis incorporated 21 studies [8, 9, 12, 21-38] published between 2014 and 2025, comprising eight RCTs and 13 observational cohort studies. The included studies were geographically diverse, predominantly from Western Europe (n=9), followed by East Asia (n=4), transatlantic collaborations between the United States and Europe (n=3), North America (n=2), and international or multicontinental cohorts (n=2). A single study originated from Central/Eastern Europe (Czech Republic, n=1).

Study Characteristics

Across 21 studies, 8,304 patients were included. The details of the included studies are given in Table 2.

**Table 2:** Baseline characteristics of the included studies Summary of patient demographics, atrial fibrillation subtypes, risk scores, comorbidities, and prior cardiac procedures across randomised controlled trials, registries, and cohort studies comparing left atrial appendage occlusion (LAAO) devices and oral anticoagulation (OAC). Data are presented as mean ± SD or n (%). RCT, randomised controlled trial; OAC, oral anticoagulation; LAAC/LAAO, left atrial appendage closure/occlusion; TAVR, transcatheter aortic valve replacement; PCI, percutaneous coronary intervention; CABG, coronary artery bypass grafting; ACS, acute coronary syndrome.

Study ID	Study type	Registration number	Region	Comparison	Number of participants	Age	Female	Atrial fibrillation types				CHA2DS2-VASc score	HAS-BLED score	Comorbidities				ACS	Prior Procedures	
								Paroxysmal	Persistent	Long-standing persistent	Permanent			Prior stroke/TIA	Diabetes	Heart failure	Hypertension		Prior PCI	Prior CABG
Gloekler, 2020 [37], APPLY study	Retrospective cohort	NCT02787525	Zurich	Amplatzer	500	73.9±10.1	155 (31)	-	-	-	-	4.3 ± 1.7	3.0 ± 1.1	-	-	-	-	-	-	-
				Warfarin	500	74.1±10.3	155 (31)	-	-	-	-	4.3 ± 1.8	2.9 ± 0.4	-	-	-	-	-	-	-
Osmancik, 2022 [8], PRAGUE-17	RCT	NCT02426944	Czech Republic (10 centres)	LAAC (Amulet 61.3%, Watchman 35.9%, Watchman-FLX in 2.8%)	201	73.4 ± 6.7	67 (33.3)	53 (26.4)	47 (23)	18 (9)	83 (41)	4.7 ± 1.5	3.1 ± 0.9	66 (90.4)	73 (36.3)	88 (43.8)	186 (92.5)	-	-	-
				DOAC	201	73.2 ± 7.2	155 (35.3)	67 (33.3)	46 (23)	16 (8)	72 (36)	4.7 ± 1.5	3.0 ± 0.9	63 (91.3)	90 (44.8)	90 (44.8)	186 (92.5)	-	-	-
Reddy, 2014 [9], PROTECT AF	RCT	NCT00129545	United States and Europe (59 centres)	Watchman 2.5	463	71·7 ± 8·8	137 (29.5)	200 (43.1)	97 (21)	-	160 (35)	-	-	82 (18)	113 (24)	124 (27)	413 (89)	-	-	-
				Warfarin	244	72·7 ± 9·2	73 (29.9)	99 (40.5)	50 (20)	-	93 (38)	-	-	49 (20)	72 (30)	66 (27)	220 (90)	-	-	-
Kapadia, 2023 [12], WATCH-TAVR	RCT	NCT03173534	North America (34 centres)	TAVR + Watchman 2.5	177	80.8±7.8	69 (39)	84 (47.4)	31 (18)	-	41 (23)	4.8 ± 1.2	3.0 ± 1.1	23 (13.1)	76 (42.9)	140 (79)	163 (92.1)	-	-	-
				TAVR + Warfarin	172	81.5±6.4	66 (38.4)	83 (48.3)	40 (23)	21 (12)	33 (19)	4.9 ± 1.2	3.0 ± 1.2	23 (13.5)	68 (39.5)	145 (84)	158 (91.9)	-	-	-
Holmes, 2014 [21], PREVAIL	RCT	NCT01182441	United States and Europe (41 centres)	Watchman 2.5	269	74.0 ± 7.4	87 (32.3)	131 (48.7)	85 (32)	-	42 (16)	3.8 ± 1.2		74 (27.5)	91 (33.8)	63 (23.4)	-	-	-	-
				Warfarin	138	74.9 ± 7.2	35 (25.4)	71 (51.4)	39 (28)	-	22 (16)	3.9 ± 1.2		39 (28.3)	41 (29.7)	32 (23.2)	-	-	-	-
Saliba, 2025 [22], OPTION	RCT	NCT03795298	International (106 centres)	Watchman FLX	803	69.6 ± 7.4	283 (35.2)	477 (59.4)	326 (41)	-	-	3.5 ± 1.3	1.2 ± 0.9	80 (10)	-	161 (20)	-	45 (6)	241 (30)	96 (12)
				DOAC	797	69.5 ± 7.9	263 (32.9)	501 (62.9)	296 (37)	-	-	3.6 ± 1.3	1.2 ± 0.8	92 (12)	-	166 (21)	-	44 (6)	262 (33)	82 (10)
Kleinecke, 2019 [23]	Prospective cohort	-	Germany and Switzerland	Watchman 2.5	266	75.3 ± 7.8	85 (31.9)	-	-	-	-	4.5 ± 1.7	3.2 ± 1.0	75 (28)	82 (31)	76 (29)	243 (91)	-	131 (49)	114 (43)
				Amulet	266	75.1 ± 9.9	87 (32.7)	-	-	-	-	4.5 ± 1.5	3.2 ± 1.0	82 (31)	74 (28)	71 (27)	244 (92)	-	134 (50)	122 (46)
Saad, 2021 [26]	Registry	-	Germany	Watchman 2.5	113	77 ± 5.1	43 (38)	-	-	-	-	4 ± 1.5	3 ± 1.0	10 (9)	35 (31)	53 (47)	100 (89)	-	-	-
				Amulet	113	78 ± 4.0	43 (38)	-	-	-	-	4 ± 1.5	3 ± 1.0	7 (6)	37 (33)	55 (49)	101 (89)	-	-	-
Galea, 2024 [25], SWISS APERO	RCT	NCT03399851	Switzerland	Watchman 2.5 (till 2019), FLX (after 2019)	110	77.3 ± 8.4	33 (30)	44 (40)	-	-	-	4.4 ± 1.4	3.2 ± 1.0	42 (38)	34 (31)	5 (5)	90 (82)	-	14 (13)	2 (2)
				Amplatzer	111	76.5 ± 7.1	32 (28.8)	43 (38.7)	-	-	-	4.2 ± 1.4	3.1 ± 0.8	45 (41)	24 (22)	5 (5)	87 (78)	-	10 (9)	3 (3)
Lakkireddy, 2021 [24], Amulet IDE	RCT	NCT02879448	United States and International (108 centres)	Watchman 2.5	915	74.0 ± 8.0	385 (41.2)	528 (57.7)	250 (27)	-	156 (17)	4.5 ± 1.3	-	168 (18.0)	461 (50)	-	-	136 (14.6)	-	-
				Amulet	916	73.8 ± 7.9	365 (38.7)	509 (55.5)	277 (30)	-	157 (17)	4.7 ± 1.4	-	188 (19.9)	504 (55)	-	-	149 (15.8)	-	-
Kretzler, 2022 [27]	Prospective cohort	DRKS00023803	Germany	Watchman 2.5	389	75.1 ± 8.5	37 (9.5)	171 (43.9)	70 (18)	-	148 (38)	4.1 ± 1.5	3.6 ± 1.1	117 (30)	165 (42)	95 (24)	369 (95)	-	88 (23)	-
				Amulet	93	74.4 ± 8.7	13 (13.9)	39 (41.9)	21 (23)	-	30 (32)	4.2 ± 1.5	3.3 ± 1.2	26 (28)	34 (37)	11 (12)	88 (95)	-	25 (27)	-
Cruz-Gonzalez, 2014 [29]	Prospective cohort	-	Spain	Watchman 2.5	10	77.49 ±54	5 (50)	-	-	-	-	3.7 ± 0.8	4.7 ± 1.3	3 (30)	-	-	-	-	-	-
				Amplatzer	21	76.2 ± 7.9	8 (38.1)	-	-	-	-	3.4 ± 0.7	4.3 ± 6.7	8 (38)	-	-	-	-	-	-
Kim, 2016 [30]	Registry	-	Korea	Watchman 2.5	46	65.6 ± 8.8	19 (41.3)	9 (19.5)	26 (57)	-	11 (24)	4.1 ± 1.7	2.8 ± 1.2	20 (43)	20 (43)	24 (52)	35 (76)	-	18 (39)	-
				Amplatzer	50	64.7 ± 10.0	18 (36)	13 (26)	23 (46)	-	14 (28)	3.6 ± 1.6	2.7 ± 1.3	22 (44)	17 (34)	15 (30)	33 (66)	-	19 (38)	-
Figini, 2016 [31]	Retrospective cohort	-	Italy	Watchman 2.5	66	72 ± 8	28 (42.4)	-	-	-	-	3.8 ± 1.6	3.4 ± 1.6	19 (29)	15 (23)	-	-	-	-	-
				Amplatzer	99	72 ± 9	28 (28.2)	-	-	-	-	4 ±1.7	3.7 ± 1.5	29 (29)	31 (31)	-	-	-	-	-
Fastener, 2018 [32]	Retrospective cohort	-	Germany	Watchman 2.5	154	75.2 ± 2.8	49 (31.8)	61 (39.6)	41 (27)	-	45 (29)	4.5 ± 0.1	3.6 ± 0.2	44 (28.6)	52 (33.8)	47 (30.5)	148 (96.1)	-	98 (63.6)	-
				Amplatzer	35	77.1 ± 9.7	13 (37.1)	17 (48.5)	4 (11)	7 (20)	14 (40)	4.0 ± 1.4	3.7 ± 1.0	8 (22.9)	14 (40.0)	8 (22.9)	34 (97.1)	-	19 (54.3)	-
Mansour, 2021 [28]	RCT	-	France	Watchman 2.5	108	76 ± 6.9	6 (24)	-	-	-	-	3.9 ± 1.3	4.2 ± 0.9	12 (48)	6 (6)	1 (1)	17 (16)	-	-	-
				Amplatzer	92	75 ± 7.4	6 (23)	-	-	-	-	3.9 ± 1.2	4.1 ± 1.2	12 (13)	5 (5)	1 (1)	21 (23)	-	-	-
Cheung, 2019 [33]	Prospective cohort	-	Hongkong	Watchman 2.5	67	72.6+7.9	21 (31.3)	19 (28.3)	-	-	47 (70)	-	-	20 (29.8)	9 (13)	52 (78)	-	-	12 (17.9)	2 (3)
				Amplatzer	77	71 ± 8.6	27 (35)	23 (29.8)	5 (6.4)	-	54 (70.1)	-	-	31 (40.2)	15 (19.4)	59 (76.6)	-	-	13 (16.8)	3 (3.8)
				Amulet/LAmbre	18	71 ± 8.6	4 (22.2)	-	-	-	-	-		10 (55.5)	7 (38.8)	12 (66.6)	-	-	4 (22)	-
Ledwoch, 2020 [34], LAARGE registry	Registry	NCT02230748	Germany (38 centres)	Watchman 2.5	278	77 ± 5.0	105 (37.7)	-	-	-	-	4.4 ± 1.5	3.9 ± 1	66 (24)	78 (28)	84 (30)	261 (94)	-	129 (46)	-
				Amplatzer/Amulet	340	77 ± 5.1	134 (39.4)	-	-	-	-	4.6 ± 1.6	3.9 ± 1.2	107 (31)	130 (38)	87 (26)	313 (92)	-	156 (46)	-
Chiu, 2022 [35]	Retrospective cohort	-	Taiwan	Watchman 2.5	56	72.7 ± 8.3	24 (42.8)	-	-	-	-	4.2 ± 1.4	3.5 ± 1.7	22 (39)	18 (32)	10 (18)	42 (75)	-	30 (54)	-
				Amplatzer/Amulet	56	71.1 ± 11.4	20 (35.7)	-	-	-	-	3.9 ± 1.8	3.3 ± 1.9	15 (27)	15 (27)	8 (14)	37 (66)	-	27 (48)	-
Fan, 2025 [36], LAMax Trial	RCT	NCT04429646	China (multicentre)	Watchman 2.5	118	69.4 ± 8.4	39 (33.1)	39 (33)	77 (65)	2 (2)	-	4.0 ± 1.4	2.7 ± 0.9	52 (44)	29 (25)	114 (97)	78 (66)	1 (1)	9 (8)	-
				LAmax	118	70.0 ± 8.5	50 (42.4)	42 (35.5)	76 (64)	0 (0)	-	4.1 ± 1.4	2.8 ± 1.1	59 (50)	19 (16)	114 (97)	80 (68)	3 (3)	11 (9)	-
Chen, 2019 [38]	Prospective Cohort	-	Germany	Watchman 2.5	36	75.3 ± 8.8	10 (27.7)	8 (22.2)	22 (61)	-	-	3.6 ± 1.5	3.9 ± 1.1	6 (17)	10 (28)	5 (13.8)	33 (92)	-	15 (42)	1 (3)
				Amulet	30	77.6 ± 8.9	15 (50)	14 (46.6)	16 (53)	-	3 (10)	3.9 ± 1.5	3.8 ± 1.0	4 (13)	9 (30)	15 (50)	22 (73)	-	15 (50)	5 (17)
				LAmbre	74	76.0 ± 7.9	25 (33.7)	-	-	-	-	3.9 ± 1.5	4.1 ± 1.0	16 (22)	22 (30)	0 (0)	63 (85)	-	31 (42)	12 (16)

**Figure 2:**
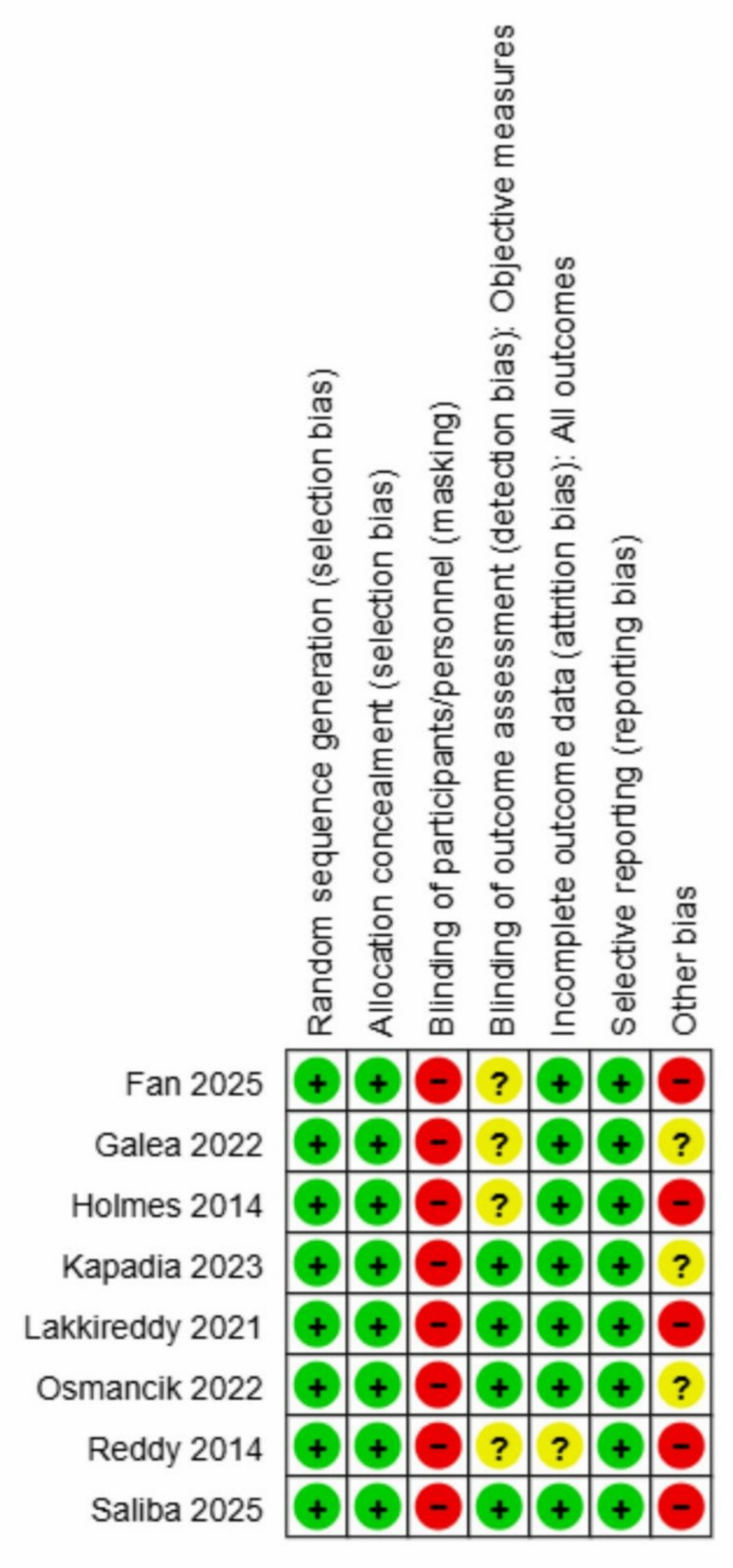
Risk of bias assessment for randomised controlled trials (Top) Individual study assessment using the Cochrane Risk of Bias 2 (RoB 2) tool across five domains; (Bottom) Summary distribution showing percentage of low risk (green), some concerns (yellow), and high risk (red) across all trials. References: Fan 2025 [36], Galea 2022 [25], Holmes 2014 [21], Kapadia 2024 [12], Lakkireddy 2021 [24], Osmancik 2022 [8], Reddy 2014 [9], Saliba 2025 [22]

RCT: Eight RCTs (PREVAIL [9], PROTECT AF [8], PRAGUE-17 [12], WATCH-TAVR [23], OPTION [21], Amulet IDE [22], SWISS-APERO [24], Fan et al. 2025 [25]) demonstrated low risk of bias for randomisation and allocation concealment, as central electronic systems or computer-generated schemes with stratification were consistently applied. Independent clinical event committees and core laboratories were widely employed, minimising detection bias. Adherence to assigned interventions was well maintained, with minimal crossover. However, all trials were open-label, introducing high performance bias. Imaging-based endpoints (particularly in SWISS-APERO [24] and Fan et al. (2025) [25]) were prone to detection bias due to visible device characteristics. Attrition bias was generally low, with high follow-up completeness and transparent reporting, except for PROTECT AF [8], which showed a moderate risk due to differential withdrawal rates. Selective reporting was consistently low risk, with prespecified endpoints reported. The most frequent limitation was “other bias” related to industry involvement. PREVAIL [9], PROTECT AF [8], OPTION [18], Amulet IDE [20], and WATCH-TAVR [17] were industry-sponsored with frequent financial ties among investigators. Although adjudication and monitoring were independent, sponsor involvement in study design and analysis posed a high risk. PRAGUE-17 [12] and SWISS-APERO [21], which benefitted from partial public or academic sponsorship, showed comparatively lower concerns, while Fan et al. (2025) [32] faced residual detection bias due to unblinded imaging outcomes. Figure 2 shows the risk of bias assessment for the RCTs.

Observational studies: Thirteen observational studies (registries and cohort designs) achieved moderate-to-high methodological quality (NOS 7-9/9). Details are shown in Table 2. Strengths included consecutive patient enrolment, real-world registry data, and systematic imaging follow-up. Mansour et al. (2021) [31] achieved the highest score (9/9) due to its prospective design, pseudo-randomised device allocation as reported by the study, and 12-month multimodality follow-up. Similarly, Kleinecke et al. (2020) [32] also scored 9/9, benefitting from prospective registry enrolment, device type recorded at the institutional level, independent clinical event adjudication, 1:1 propensity-score matching, complete clinical follow-up, and adjudicated endpoints. Other high-quality studies (Saad et al. (2021) [35], Kim et al. (2016) [34], Cheung et al. (2019) [29], Ledwoch et al. (2017) [36], and Chiu et al. (2021) [37]) reported long-term follow-up (≥12-28 months) and high completeness. Figini et al. (2016) [33] and Gloekler et al. (2020) [38] scored 7/9, with limitations related to operator-dependent device allocation, incomplete imaging follow-up, or short follow-up duration. Lower-scoring studies included Kretzler et al. (2022) [30], Gonzalez et al. (2014) [26], Chen et al. (2019) [28], and Fastner et al. (2018) [27] (6/9), limited by short follow-up (≤6 months) and lack of propensity matching, increasing selection bias. Sensitivity analyses excluding lower-quality (≤6/9) studies are acknowledged as a limitation. Across registries, comparability between device cohorts remained limited as device allocation was operator-dependent, representing a consistent confounding factor and a limiting factor. Matching quality was assessed using standardised mean differences when reported. Lower NOS comparability scores reflected inadequate adjustment. Follow-up adequacy was judged relative to each endpoint.

**Table 3:** The Newcastle-Ottawa Scale (NOS) assessment of quality across 13 observational studies Higher scores indicate better methodological quality. Studies scoring ≥7 were considered high quality. References: [23, 26-35, 37, 38]

Study	Year	Representativeness of the exposed cohort	Selection of a non-exposed cohort	Ascertainment of exposure	Outcome not present at start	Comparability of cohorts	Assessment of outcome	Follow-up long enough	Adequacy of follow-up	Quality score
Saad [26]	2021	★	★	★	★	★★	★	★	★	9/9
Kretzler [27]	2022	★	★	★	★	☆	★	☆	★	6/9
Mansour [28]	2021	★	★	★	★	★★	★	★	★	9/9
Gonzalez [29]	2014	★	★	★	★	☆	★	☆	★	6/9
Kim [30]	2016	★	★	★	★	☆	★	★	★	7/9
Figini [31]	2016	★	★	★	★	☆	★	★	★	7/9
Fastner [32]	2018	★	★	★	★	☆	★	☆	★	6/9
Cheung [33]	2019	★	★	★	★	☆	★	★	★	7/9
Ledwoch [34]	2017	★	★	★	★	☆	★	★	★	7/9
Chiu [35]	2022	★	★	★	★	☆	★	★	★	7/9
Chen [38]	2019	★	★	★	★	☆	★	☆	★	6/9
Gloekler [37]	2020	★	★	★	★	☆	★	☆	★	7/9
Kleinecke [23]	2020	★	★	★	★	★★	★	★	★	9/9

LAAO versus OAC

**Figure 3:**
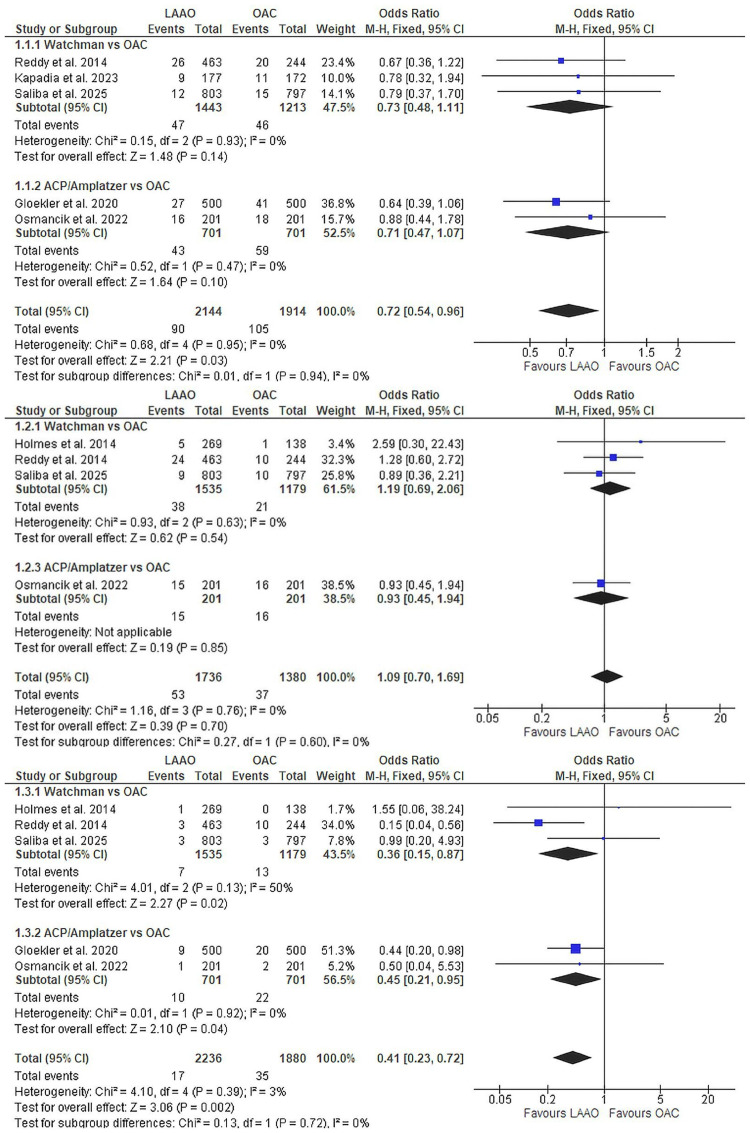
Primary efficacy outcomes comparing LAAO versus OAC (1.1) Transient ischaemic attack: five studies, n=4,058, OR 0.72 (95% CI 0.54-0.96), p=0.03; (1.2) Ischaemic stroke: four studies, n=3,116, OR 1.09 (95% CI 0.70-1.69), p=0.70. (1.3); Haemorrhagic stroke: six studies, n=4,116, OR 0.41 (95% CI 0.23-0.72), p=0.002. LAAO, left atrial appendage occlusion; OAC, oral anticoagulation; OR, odds ratio; CI: confidence interval References: [8-9, 12, 21, 23, 38]

Efficacy outcomes: Primary efficacy outcomes comparing LAAO devices collectively with oral anticoagulation are presented in Figure 3. LAAO significantly reduced TIAs by 28% (OR 0.72, 95% CI 0.54-0.96, p=0.03; five studies [8, 12, 21, 23, 38], n=4,058; Figure 3A). But subgroup classification among Watchman and ACP/Amplatzer groups showed non-significance. Ischaemic stroke rates were equivalent between platforms (OR 1.09, 95% CI 0.70-1.69, p=0.70; four studies [8-9, 12, 21], n=3,116, I²=0%; Figure 3), confirming non-inferiority of either LAAO or OACs. Haemorrhagic stroke showed the most pronounced benefit, with a 59% reduction favouring LAAO (OR 0.41, 95% CI 0.23-0.72, p=0.002, I²=3%; five studies [8-9, 12, 21, 38], n=4,116; Figure 3). Subgroup classification also showed significant superiority of Watchman (OR 0.36, 95% CI 0.15-0.87, p=0.02, I²=50%; Figure 3) and ACP/Amplatzer (OR 0.45, 95% CI 0.21-0.95, p=0.04, I²=0%; Figure 3C) over OACs. Systemic embolism events were rare, with no significant difference between Watchman and OACs (warfarin and non-VKAs; Supplementary Material 1). However, when only warfarin studies were kept as a comparator, the Watchman group had significantly fewer systemic embolism events. (OR 0.17, 95% CI 0.03-0.86, p=0.03, I²=61%; Supplementary Material 1).

Safety outcomes: Major bleeding showed a non-significant trend favouring LAAO (OR 0.80, 95% CI 0.61-1.05, p=0.11; four studies, n=3,058), with detailed analysis in Supplementary Material 2.

**Figure 4:**
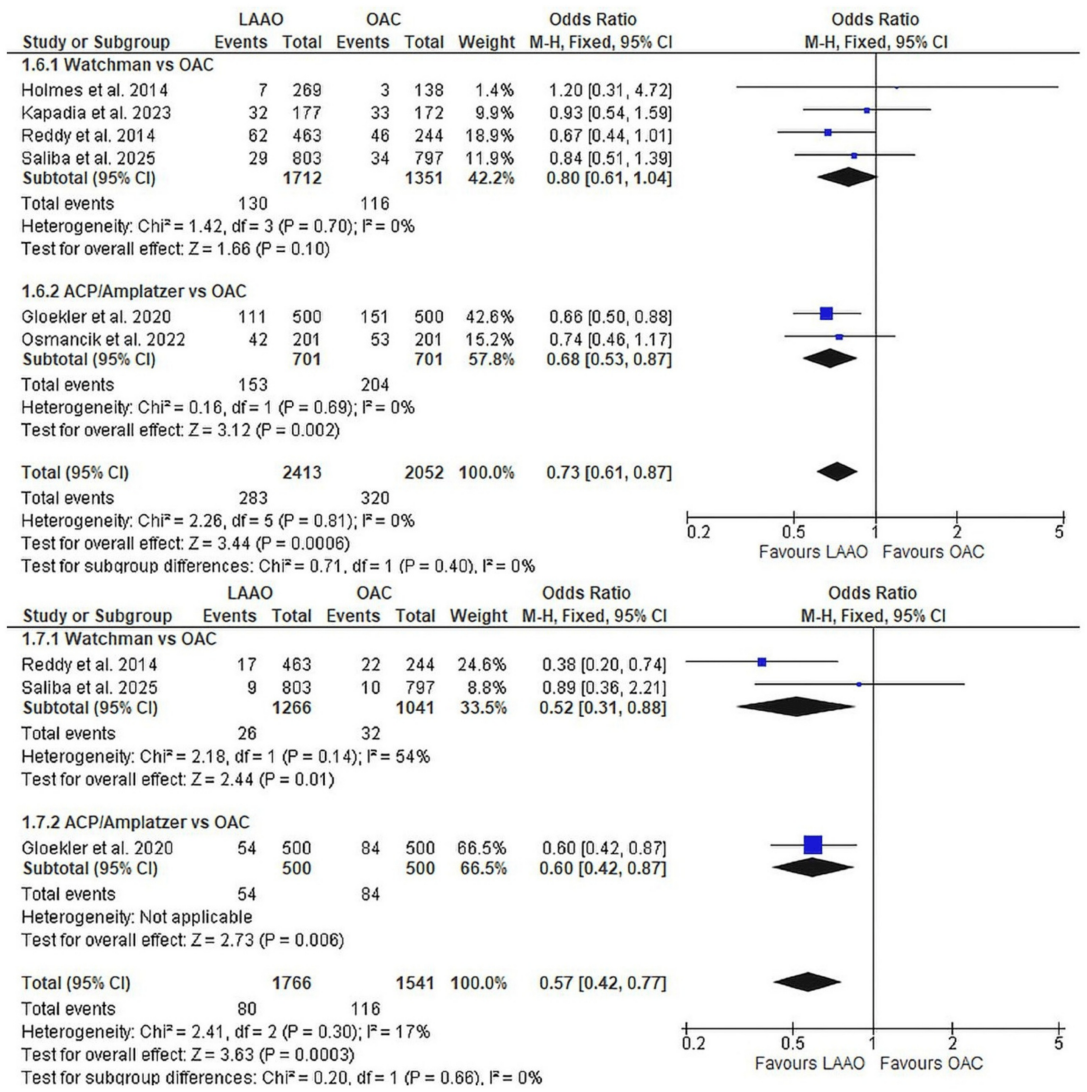
Mortality outcomes comparing LAAO versus OAC (1.6) All-cause mortality: six studies, n=4,465, OR 0.73 (95% CI 0.61-0.87), p<0.001; (1.7) Cardiovascular mortality: three studies, n=3,307, OR 0.57 (95% CI 0.42-0.77), p<0.001. LAAO, left atrial appendage occlusion; OAC, oral anticoagulation; OR, odds ratio; CI: confidence interval References: [8-9, 12, 21, 23, 38]

Mortality outcomes: Mortality outcomes strongly favoured LAAO (Figure 4). All-cause mortality was reduced by 27% (OR 0.73, 95% CI 0.61-0.87, p<0.001, I^2^=0%; six studies [8-9, 12, 21, 23, 38], n=4,465; Figure 4). While the combined analysis was significantly favouring LAAOs, the subgroup classification was only found to be significant in the ACP/Amplatzer group over OACs. Cardiovascular mortality demonstrated an even greater 43% reduction with LAAO (OR 0.57, 95% CI 0.42-0.77, p<0.001, I^2^=17%; three studies [8, 21, 38], n=3,307; Figure 4).

Watchman versus other LAAO devices

**Figure 5:**
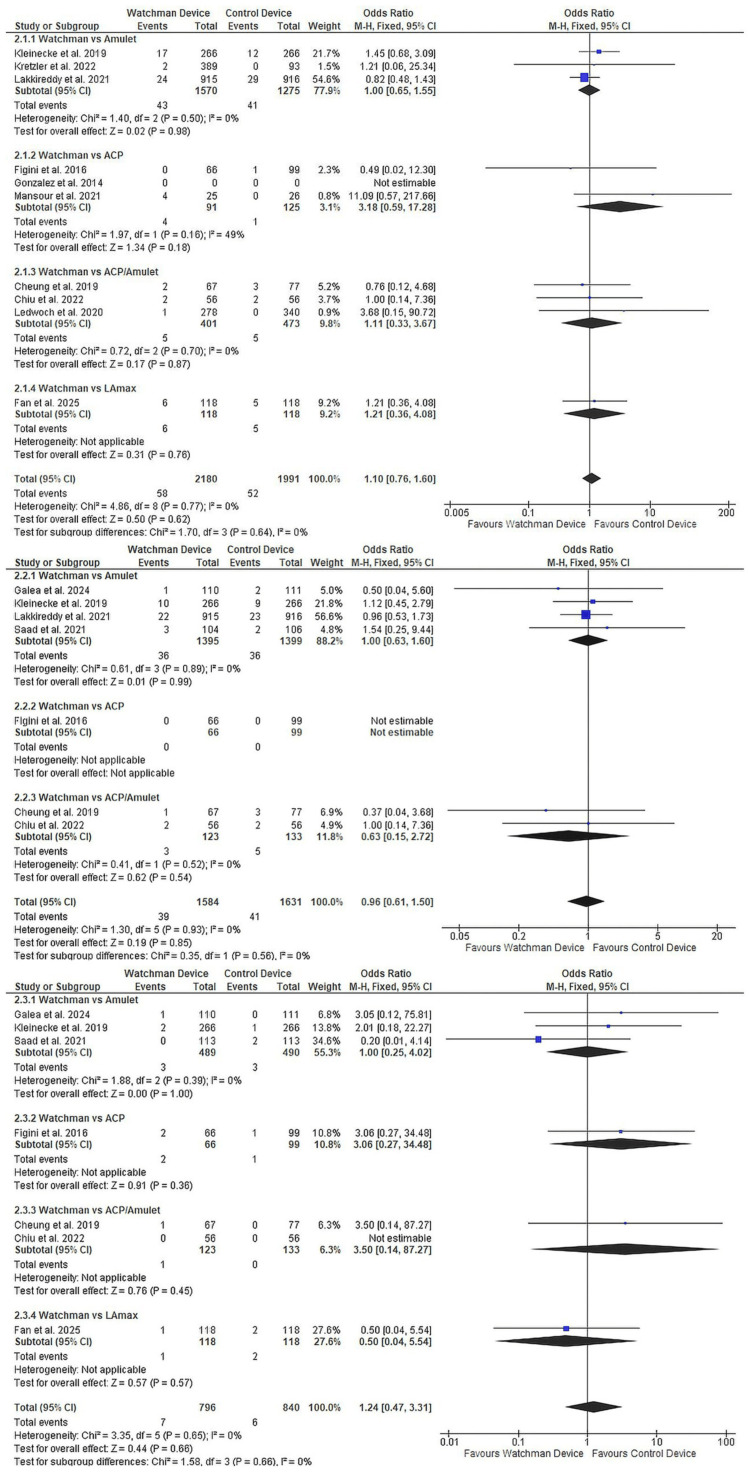
Primary efficacy comparing Watchman versus other LAAO devices (2.1) TIA: 10 studies, n=4,171, OR 1.10 (95% CI 0.76-1.60), p=0.62; (2.2) Ischaemic stroke: seven studies, n=3,215, OR 0.96 (95% CI 0.61-1.50), p=0.85; (2.3) Haemorrhagic stroke: seven studies, n=1,636, OR 1.24 (95% CI 0.47-3.31), p=0.66. LAAO, left atrial appendage occlusion; OAC, oral anticoagulation; OR, odds ratio; CI: confidence interval; TIA: transient ischemic attack References: TIA: 10 studies [22, 25, 26, 29-33, 36, 37]; ischaemic stroke: seven studies [22, 24, 29, 32, 33, 35, 37]; haemorrhagic stroke: seven studies [24, 25, 29, 32, 33, 35, 37]

Efficacy outcomes: Device comparison efficacy outcomes are displayed in Figure 5. No significant differences were observed between Watchman and other devices for TIA (OR 1.10, 95% CI 0.76-1.60, p=0.62; 10 studies [22, 25, 26, 29-33, 36, 37], n=4,171; Figure 5), ischaemic stroke (OR 0.96, 95% CI 0.61-1.50, p=0.85; seven studies [22, 24, 29, 32, 33, 35, 37], n=3,215; Figure 5), or haemorrhagic stroke (OR 1.24, 95% CI 0.47-3.31, p=0.66; 7 studies [24, 25, 29, 32, 33, 35, 37], n=1,636; Figure 5). Heterogeneity was found to be low across all comparisons. The wide CI for haemorrhagic stroke reflects only 13 total events. Systemic embolism comparisons showed similar equivalence (Supplementary Material 1).

**Figure 6:**
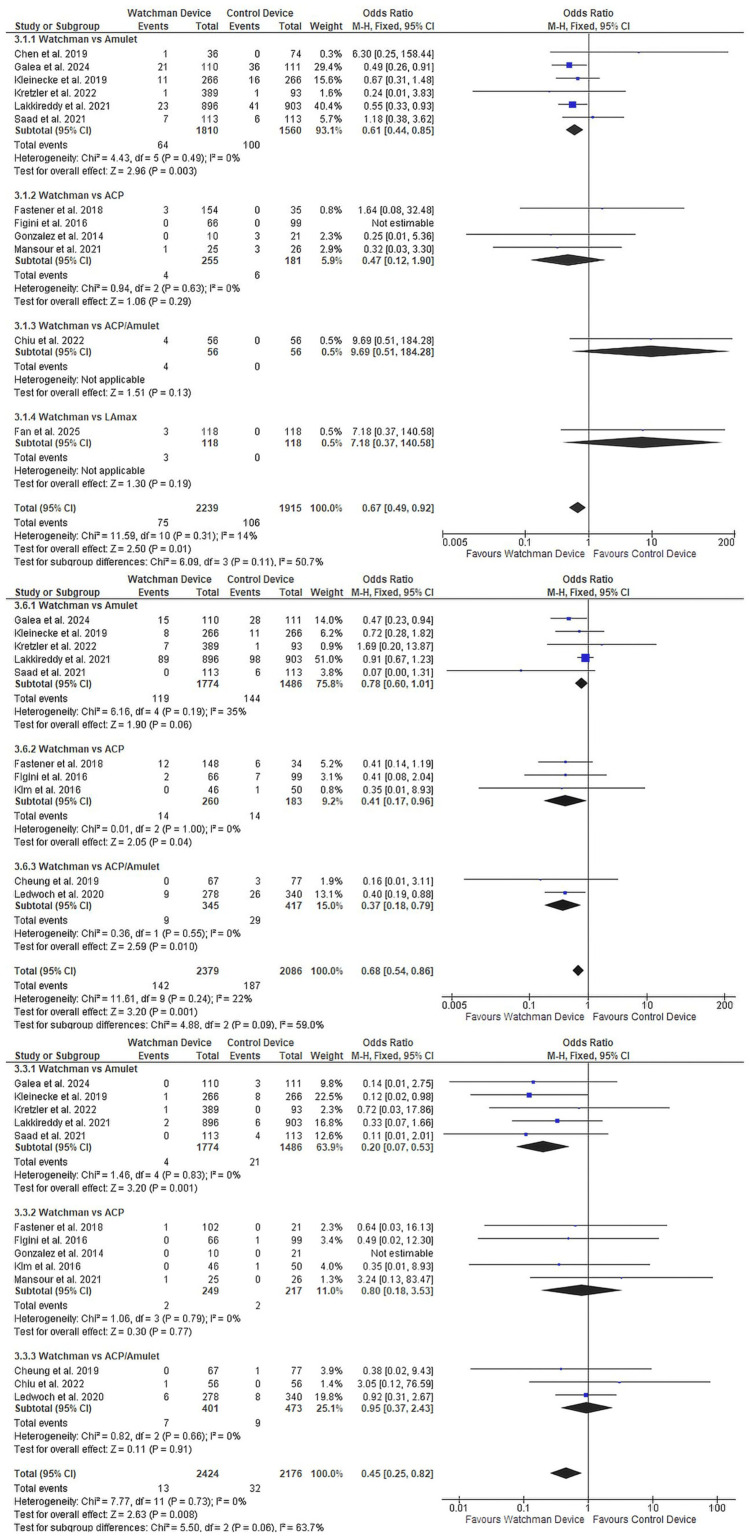
Key safety outcomes favoring Watchman (3.1) Device-related complications: 12 studies, n=4,154, OR 0.67 (95% CI 0.49-0.92), p=0.01; (3.3) Device embolisation: 13 studies, n=4,600, OR 0.45 (95% CI 0.25-0.82), p=0.009; (3.6) Procedural bleeding: 10 studies, n=4,465, OR 0.68 (95% CI 0.54-0.86), p=0.001. OR, odds ratio; CI, confidence interval References: Device-related complications: 12 studies [22, 24-28, 30-33, 35, 37]; Device embolisation: 13 studies [22, 24, 26, 27, 29-37]; Procedural bleeding: 10 studies [22, 24, 27, 29, 30, 32-36]

Safety outcomes: Key safety outcomes significantly favoured Watchman (Figure 6). Device-related complications were reduced by 33% (OR 0.67, 95% CI 0.49-0.92, p=0.01; 12 studies [22, 24-28, 30-33, 35, 37], n=4,154; Figure 6), with the benefit most pronounced versus Amulet (OR 0.61, p=0.003). Device embolisation risk was 55% lower with Watchman overall (OR 0.45, 95% CI 0.25-0.82, p=0.009; 13 studies [22, 24, 26, 27, 29-37], n=4,600; Figure 6), particularly most pronounced compared with Amulet (OR 0.20, p=0.001). Procedural bleeding was also significantly reduced by 32% overall (OR 0.68, 95% CI 0.54-0.86, p=0.001; 10 studies [22, 24, 27, 29, 30, 32-36], n=4,465; Figure 6) as well as all subgroup comparisons.

Additional significant safety outcomes are detailed in Supplementary Materials 3-7. Pericardial effusion requiring intervention was lower with Watchman (OR 0.74, 95% CI 0.55-0.98, p=0.04; Supplementary Material 3). Major procedural complications favoured Watchman despite high heterogeneity (OR 0.71, 95% CI 0.52-0.96, p=0.03, I²=75%; Supplementary Material 6). No differences were observed for device-related thrombosis (p=0.78), major vascular complications (p=0.82), or procedure-related stroke (p=0.95; Supplementary Materials 4, 5, 7).

Peridevice Leak Analysis

**Figure 7:**
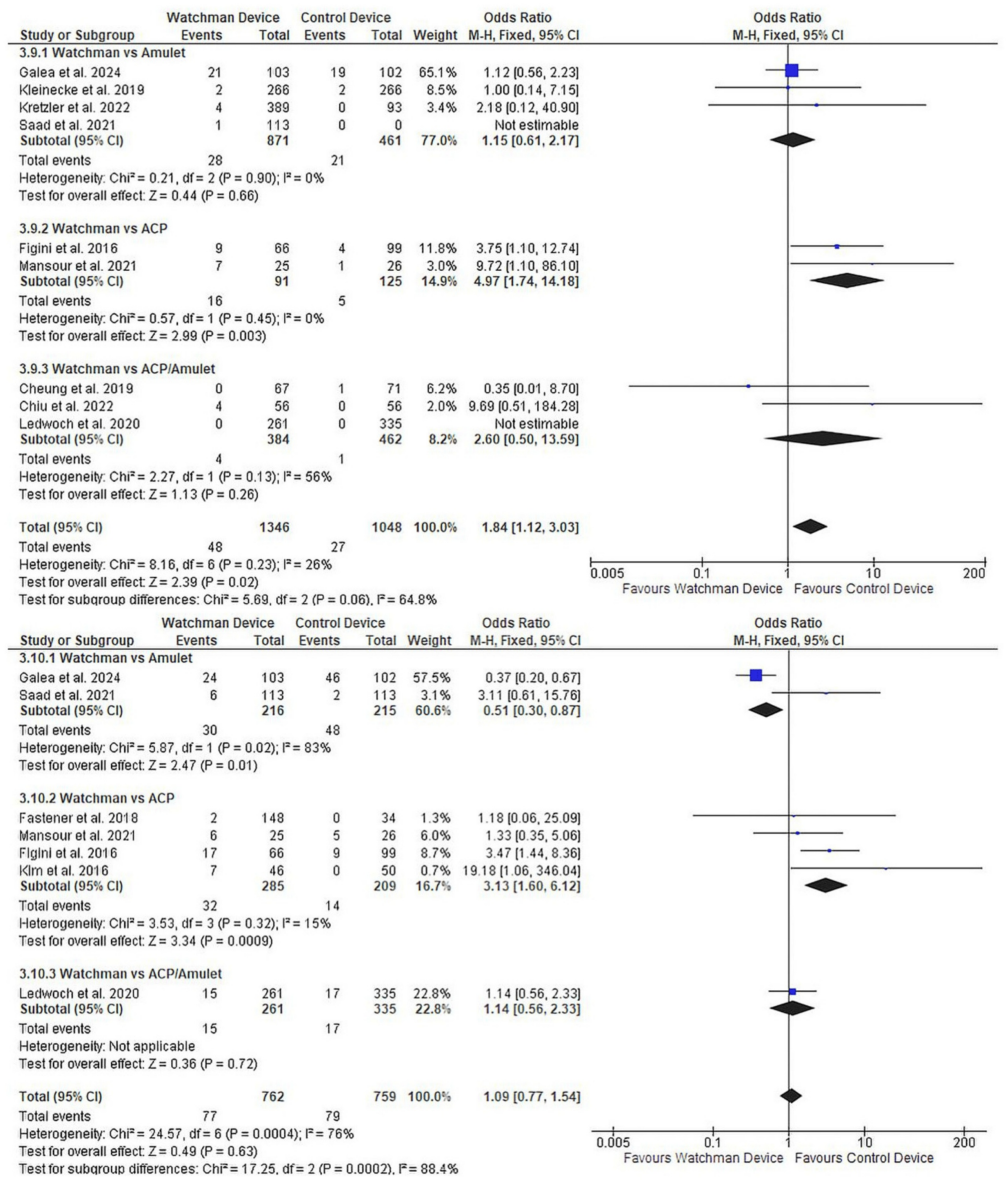
Peridevice leak comparison between devices (3.9) Major leak ≥5mm: nine studies, n=2,394, OR 1.84 (95% CI 1.12-3.03), p=0.02, favouring other devices; (3.10) Minor leak <5mm: seven studies, n=1,521, OR 1.09 (95% CI 0.77-1.54), p=0.63, with device-specific variability. OR, odds ratio; CI, confidence interval References: Major leak ≥5mm: nine studies [24, 29-33, 35-37]; Minor leak <5mm: seven studies [24, 27, 31, 33-36]

Peridevice leak assessment revealed an important trade-off (Figure 7). Major leaks (≥5mm) were 84% more frequent with Watchman (OR 1.84, 95% CI 1.12-3.03, p=0.02; 9 studies [24, 29-33, 35-37], n=2,394; Figure 7), with the difference most pronounced versus ACP (OR 4.97, p=0.003). Minor leaks (<5mm) showed device-specific variability without overall difference (OR 1.09, 95% CI 0.77-1.54, p=0.63; seven studies [24, 27, 31, 33-36], n=1,521; Figure 7), with Watchman having fewer minor leaks than Amulet (OR 0.51, p=0.01) but more than ACP (OR 3.13, p<0.001).

Procedural Success and Major Bleeding

Procedural success rates exceeded 95% for all devices with no significant differences (OR 0.96, 95% CI 0.66-1.40, p=0.82; 11 studies, n=4,165; Supplementary Material 8). Major bleeding showed no difference between devices (OR 0.86, 95% CI 0.69-1.07, p=0.17; nine studies, n=4,355; Supplementary Material 1).

Mortality Outcomes

**Figure 8:**
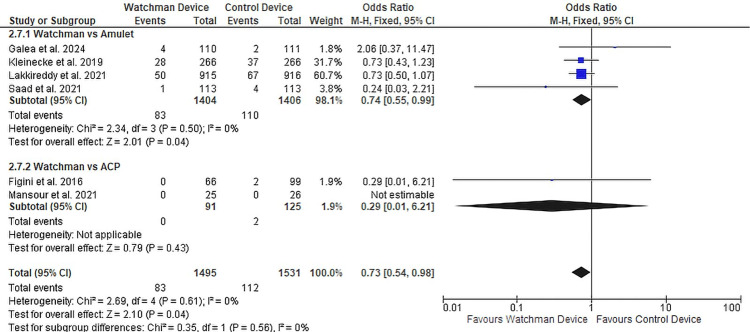
Cardiovascular mortality comparing Watchman versus other LAAO devices Six studies, n=3,026, OR 0.73 (95% CI 0.54-0.98), p=0.04, demonstrating survival benefit with Watchman. LAAO, left atrial appendage occlusion; OR, odds ratio; CI: confidence interval References: [22, 24, 31-33, 35]

Cardiovascular mortality was significantly lower with Watchman compared to other devices (OR 0.73, 95% CI 0.54-0.98, p=0.04; six studies [22, 24, 31-33, 35], n=3,026; Figure 8), primarily driven by comparison with Amulet. All-cause mortality showed a similar but non-significant trend toward benefit (OR 0.78, 95% CI 0.60-1.01, p=0.06; 13 studies, n=4,663; Supplementary Material 9).

Heterogeneity Report

Moderate to high heterogeneity was observed primarily in comparisons involving Watchman, likely reflecting the larger number of contributing studies, inclusion of both first- and second-generation devices, wider variability in operator experience, and more heterogeneous imaging follow-up protocols. In contrast, analyses of ACP, Amulet, and other devices showed low heterogeneity, largely due to fewer studies, more uniform device generations, and more consistent procedural and imaging practices within those smaller datasets.

Discussion

The present meta-analysis offers critical insights into the evolving landscape of mechanical stroke prevention strategies, providing a robust quantitative synthesis of comparative outcomes between percutaneous appendage closure and pharmacological anticoagulation while delineating important performance distinctions among available device platforms.

Comparative Effectiveness of Mechanical Versus Pharmacological Stroke Prevention

Our analysis identified a 28% relative risk reduction in transient ischaemic attack with LAAO compared to anticoagulation therapy (OR 0.72, 95% CI 0.54-0.96; P=0.026). This observation, not consistently reported in individual studies, potentially reflects fundamental differences in stroke prevention mechanisms. Whereas anticoagulation effectiveness fluctuates with medication levels and adherence patterns, appendage exclusion provides continuous protection independent of patient compliance or pharmacokinetic variability. The comparable ischaemic stroke rates between strategies (OR 1.09, 95% CI 0.70-1.69) align with contemporary trial data, including PRAGUE-17, supporting equivalence in preventing major thromboembolic events [12].

The observed reduction in intracranial bleeding events associated with LAAO (OR 0.41, 95% CI 0.23-0.72; P=0.002) represents a substantial finding. This 59% relative risk reduction aligns mechanistically with the elimination of anticoagulation as a risk factor for spontaneous haemorrhage. This magnitude of benefit corresponds closely with long-term PROTECT AF data showing an 85% haemorrhagic stroke reduction at five years [8, 39]. The mechanism underlying this protection appears straightforward, i.e., elimination of anticoagulation removes the primary risk factor for spontaneous intracranial haemorrhage.

However, it is crucial to note that most comparative data derive from trials using warfarin as the comparator. The ongoing CHAMPION-AF trial will provide important clarification on this comparison [40]. While DOACs have improved upon warfarin's bleeding profile, comprehensive network analyses confirm that even these newer agents cannot completely eliminate haemorrhagic complications [41].

The observed mortality advantages warrant particular attention, with our analysis documenting a 27% reduction in all-cause death (OR 0.73, 95% CI 0.61-0.87) and an even more pronounced 43% decrease in cardiovascular mortality (OR 0.57, 95% CI 0.42-0.77). These survival benefits exceed what would be anticipated from stroke prevention alone, suggesting additional protective mechanisms. Recent network meta-analyses corroborate these findings, with Carnicelli et al. [41] reporting consistent mortality advantages for LAAO across multiple analytical approaches. Potential explanations for enhanced survival include avoidance of both recognised and subclinical bleeding complications, elimination of medication-related adverse effects, and possible beneficial haemodynamic consequences of appendage exclusion. It can also likely reflect residual confounding, including comorbidity imbalance and selection patterns not fully captured in observational datasets. However, patients selected for LAAO in real-world practice may be systematically healthier or receive more intensive medical management than those maintained on anticoagulation.

Inter-Device Performance Comparisons and Clinical Implications

Our comprehensive device comparison analysis, encompassing over 4,600 patients across multiple studies, provides the most robust evidence to date regarding differential outcomes between leading LAAO platforms. The absence of significant efficacy differences for major endpoints, including TIA (OR 1.10, 95% CI 0.76-1.60) and ischaemic stroke (OR 0.96, 95% CI 0.61-1.50), suggests that successful appendage isolation, rather than specific device characteristics, determines thromboembolic protection. This interpretation gains support from the Amulet IDE trial's demonstration of non-inferiority between platforms for composite efficacy outcomes [22]. The design variations reflect different philosophies regarding optimal appendage closure: single versus dual-component architecture, proximal versus deep anchoring strategies, and immediate versus delayed endothelialisation patterns. Device-iteration timelines (older ACP vs contemporary Amulet/FLX) partly explain differential performance. Operator-experience effects were more pronounced in early ACP cohorts. Imaging follow-up schedules and modalities differed across platforms, influencing leak detection. Understanding these fundamental differences provides context for interpreting comparative effectiveness and safety outcomes.

Research comparing LAAO devices reveals meaningful safety distinctions between platforms. The Watchman system demonstrates superior safety profiles across multiple metrics. Price et al. (2022) found that the newer Watchman FLX significantly reduced major adverse events compared to the original Watchman 2.5 device, with lower rates of mortality (0.12% vs 0.24%), major bleeding (1.08% vs 2.05%), and device embolisation (0.02% vs 0.06%). [42] When comparing different device platforms, Qiao et al. (2022) showed Watchman devices had significantly lower rates of major procedure-related complications and device embolisation compared to Amplatzer devices (OR 0.50 and OR 0.50, respectively, for major complications and embolisation) [43]. The learning curve effect contributes to improved outcomes, as Reddy et al. (2011) demonstrated significant reductions in procedure-related safety events with increased operator experience [44]. Real-world data from Khalil et al. (2020) confirmed low complication rates with Watchman implantation, reporting overall complications of 1.9% and mortality of 0.29% [45].

An important trade-off exists regarding appendage sealing completeness. Our analysis identified an 84% increased risk of significant peridevice leak with Watchman devices (OR 1.84, 95% CI 1.12-3.03), consistent with the Amulet platform's superior complete seal rates documented in randomised trials. The dual-seal architecture of the Amulet appears to provide enhanced conformability to variable appendage anatomies. However, the clinical relevance of residual peridevice flow remains controversial, with conflicting evidence regarding associations with subsequent thromboembolic events [46]. The optimal management strategy for peridevice leaks, including the role of continued anticoagulation, requires further investigation through prospective studies with standardised imaging protocols.

Translating Evidence into Clinical Practice

These findings carry substantial implications for clinical decision-making and patient counselling. The demonstrated mortality benefits and virtual elimination of haemorrhagic stroke risk establish LAAO as a compelling therapeutic option for appropriately selected patients. Current physicians' consensus documents identify multiple scenarios where mechanical closure may be preferred, particularly for individuals with previous intracranial bleeding, recurrent haemorrhage despite optimal management, or conditions creating unacceptable bleeding hazards [47].

Emerging evidence suggests potential expansion of LAAO indications beyond traditional anticoagulation-contraindicated populations. Several ongoing investigations explore novel applications, including the OPTION trial evaluating prophylactic closure following successful rhythm control procedures and CHAMPION-AF examining LAAO as primary therapy in anticoagulation-eligible patients. The substantial mortality benefits identified in our analysis provide support for investigating these expanded indications, particularly considering the persistent challenges with long-term anticoagulation adherence [40].

Device platform selection requires careful consideration of multiple factors. Our findings suggest Watchman systems may be preferable when prioritising procedural safety, particularly for patients with elevated periprocedural risk or when performed by operators with limited experience. Recent analyses have documented strong correlations between procedural volume and outcomes, with high-volume centres achieving significantly lower complication rates [48]. Alternatively, the Amulet's superior sealing characteristics may prove advantageous for patients with complex appendage anatomy or those at highest thromboembolic risk requiring complete exclusion. The net clinical benefit of LAAO is strongest in patients with major bleeding, inability to tolerate long-term anticoagulation, or recurrent bleeding on therapy. Mortality findings should not be generalised to low-risk atrial fibrillation populations. Management of minor-moderate peridevice leaks remains uncertain and should be individualised. Device choice may depend on imaging follow-up availability. Findings generally align with guideline recommendations emphasising individualised assessment.

Economic Implications and Healthcare System Considerations

Multiple economic evaluations demonstrate favourable cost-effectiveness profiles for LAAO across different healthcare systems. Lee et al. (2016) conducted a comprehensive Markov model analysis comparing LAAO with seven pharmacological strategies for stroke prevention in non-valvular atrial fibrillation from a US perspective. Their analysis showed LAAO was cost-effective with incremental cost-effectiveness ratios of $6,298 per quality-adjusted life year (QALY) compared to warfarin, $2,447 versus clopidogrel plus aspirin, and $5,115 against aspirin alone. LAAO demonstrated dominance over other anticoagulation strategies, being cost-effective in 86.24% of Monte Carlo simulations using a $50,000/QALY threshold. [49] From a Swedish healthcare perspective, Labori et al. (2021) found LAAO cost-effective for patients with atrial fibrillation contraindicated to oral anticoagulation, reporting an incremental cost-effectiveness ratio of €4,047 per QALY gained. The intervention was dominant from a public sector perspective due to reduced long-term care costs [50].

This comprehensive meta-analysis establishes LAAO as an effective stroke prevention strategy offering meaningful mortality advantages and virtual elimination of haemorrhagic stroke risk compared to anticoagulation therapy. While different device platforms demonstrate equivalent thromboembolic protection, important safety and sealing distinctions exist that should inform individualised device selection. Economic analyses in literature predominantly assess older devices and warfarin-based comparators [49-50]; cost-effectiveness for FLX or Amulet is uncertain. Real-world value varies across public vs private payer models. Most cost projections rely on extrapolated long-term assumptions rather than observed data. The accumulated evidence supports expanding LAAO utilisation beyond traditionally anticoagulation-contraindicated populations, particularly for patients with bleeding concerns or documented non-adherence. Continued technological refinement and accumulating long-term outcome data will likely position mechanical appendage closure as an increasingly attractive first-line option for stroke prevention in appropriately selected atrial fibrillation patients.

Methodological Considerations and Research Priorities

Several methodological considerations temper the interpretation of our findings. Temporal heterogeneity in comparator anticoagulation regimens, transitioning from warfarin in early trials to DOACs in contemporary studies, introduces potential bias. Additionally, variable follow-up intervals across included studies constrain assessment of long-term device performance and late complication rates. The predominance of Wathman 2.5 over the newer system Watchman FLX restricts generalisability. Unmeasured confounders, including imaging modality, antithrombotic regimens, and operator variability, remain critical limitations.

Critical knowledge gaps requiring future investigation include extended durability assessment beyond five-year horizons, particularly regarding device integrity and late thrombosis risk. Prospective randomised comparisons between next-generation device iterations, including evaluation of under-represented platforms such as LAmbre, would inform optimal platform selection. Studies on learning curves of each platform indicate ease of use in less expert operator settings. Enhanced risk stratification tools incorporating clinical, anatomical, and procedural variables could improve patient selection precision. Standardised imaging and endpoint definitions are needed for harmonised comparative research. Integration of computational modelling and artificial intelligence technologies may further refine preprocedural planning and outcome prediction.

## Conclusions

Our study demonstrates that LAAO provides stroke-prevention efficacy equivalent to OAC while significantly reducing haemorrhagic stroke and mortality. Device-level comparisons show that Watchman is associated with fewer device-related complications, embolisation events, and procedural bleeding, although with a higher rate of major peri-device leaks compared with dual-component systems.

These findings support LAAO as an effective strategy for patients with elevated bleeding risk or limited suitability for long-term anticoagulation. Clinically meaningful differences in safety and sealing performance among devices highlight the importance of anatomy-guided device selection and standardised post-procedural imaging. Ongoing trials may substantially modify current conclusions, particularly for anticoagulation-ineligible patients.

## Figures and Tables

**Figure 9:**
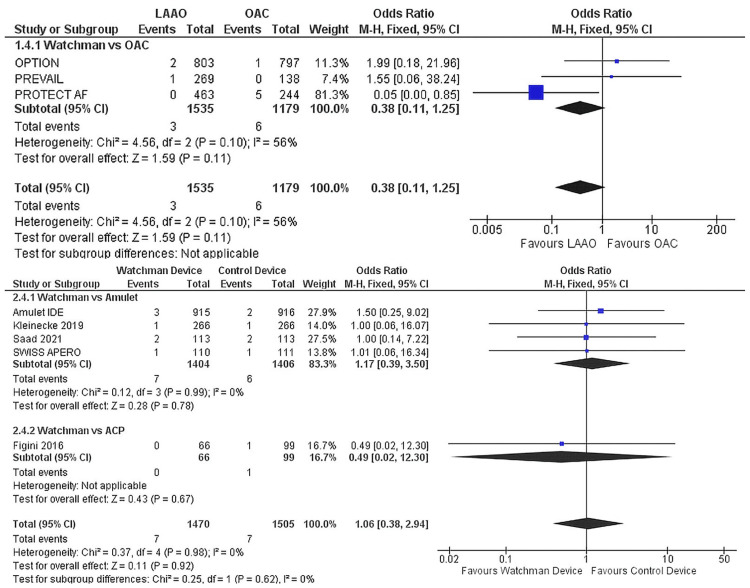
Systemic embolism; (1.4) Forest plot comparing systemic embolisation incidence between LAAO and oral anticoagulation; (2.4) Forest plot comparing systemic embolism incidence between Watchman and control devices LAAO: left atrial appendage occlusion References: OPTION [22], PREVAIL [21],  PROTECT AF [9], PRAGUE 17 [8], Amulet IDE [24], Kleinecke 2019 [23], Saad 2021 [26], SWISS APERO [25], Figini 2016 [31].

**Figure 10:**
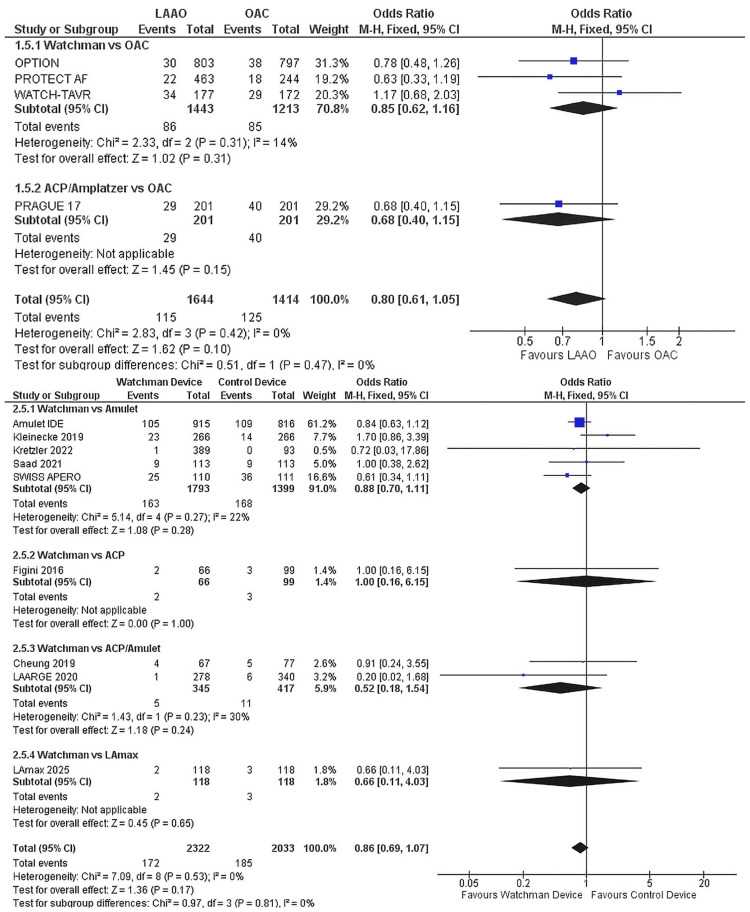
Major bleeding outcomes; (1.5) Forest plot comparing major bleeding incidence between LAAO and oral anticoagulation; (2.5) Forest plot comparing major bleeding incidence between Watchman and control devices LAAO: Left atrial appendage occlusion References: OPTION [22], PROTECT AF [9], WATCH-TAVR [12] PRAGUE 17 [8], Amulet IDE [24], Kleinecke 2019 [23], Kretzler 2022 [27], Saad 2021 [26], SWISS APERO [25], Figini 2016 [31], Cheung 2019 [33], LAARGE 2020 [34], LAmax [36].

**Figure 11:**
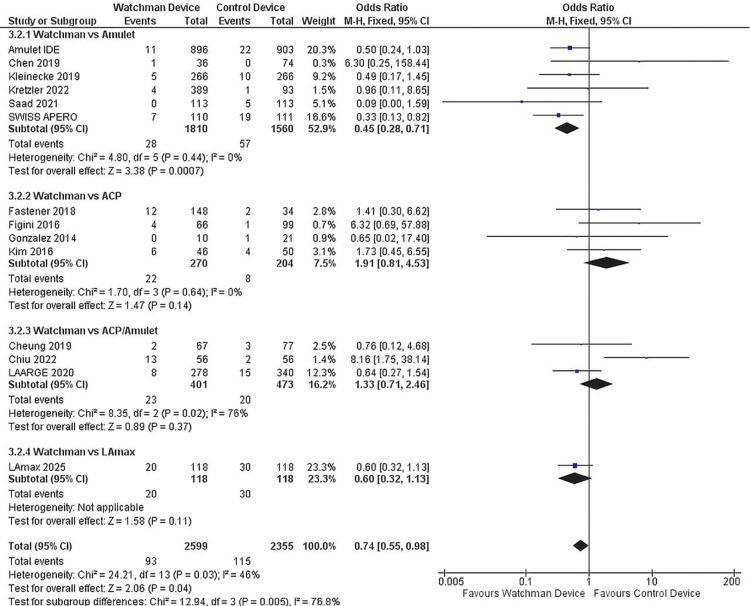
Pericardial effusion; Forest plot comparing pericardial effusion incidence between Watchman and control devices References: Amulet IDE [24], Chen 2019 [38], Kleinecke 2019 [23], Kretzler 2022 [27], Saad 2021 [26], SWISS APERO [25], Fastner [32], Figini 2016 [31], Gonzalez [29], Kim 2018 [30], Cheung 2019 [33], Chiu 2022 [35], LAARGE 2020 [34], LAmax [36].

**Figure 12:**
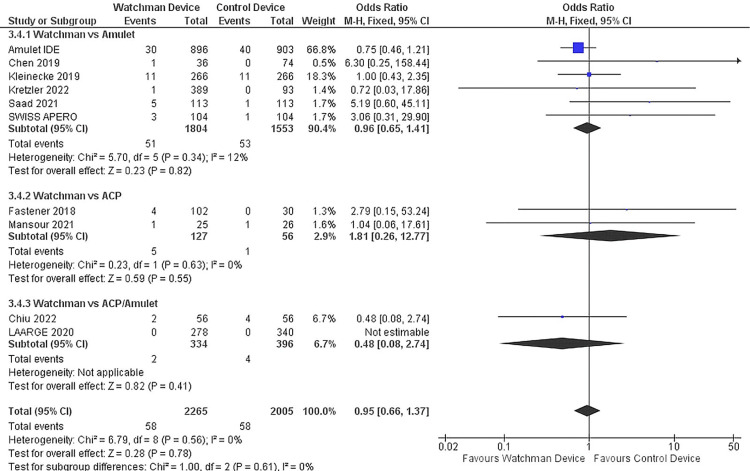
Device-related thrombosis; Forest plot comparing device-related thrombus incidence between Watchman and control devices. References: Amulet IDE [24], Chen 2019 [38], Kleinecke 2019 [23], Kretzler 2022 [27], Saad 2021 [26], SWISS APERO [25], Fastner [32], Mansour [28], Chiu [35], LAARGE 2020 [34]

**Figure 13:**
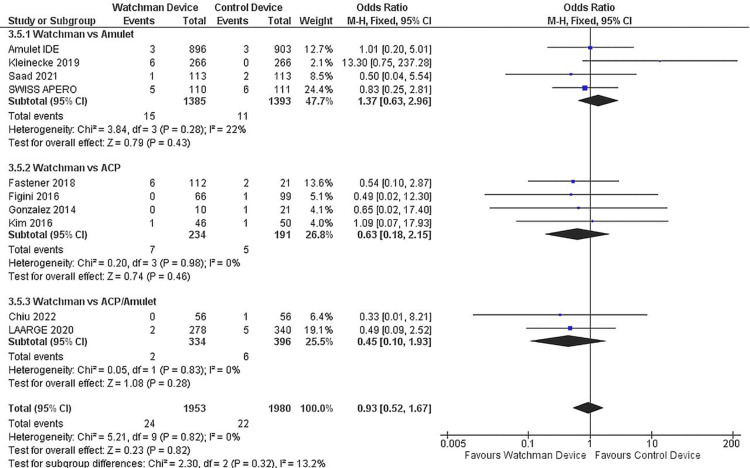
Major vascular vomplications; Forest plot comparing major vascular complications incidence between Watchman and control devices. References: Amulet IDE [24], Kleinecke 2019 [23], Saad 2021 [26], SWISS APERO [25], Fastner [32], Figini 2016 [31], Gonzalez [29], Kim 2018 [30], Chiu [35], LAARGE 2020 [34].

**Figure 14:**
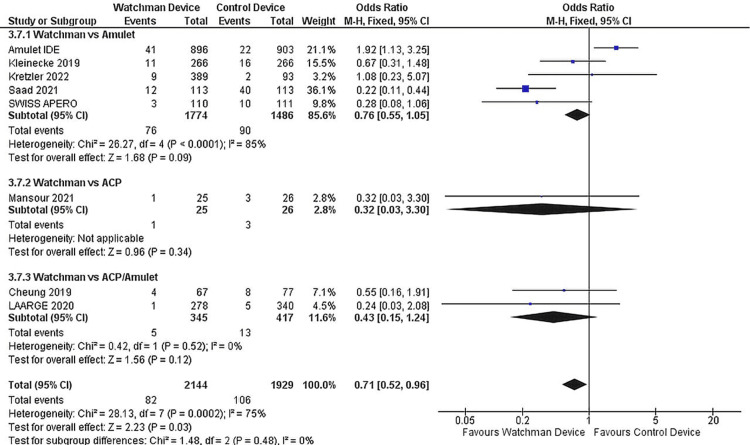
Major procedural complications; Forest plot comparing major procedural complications incidence between Watchman and control devices - Significant but high heterogeneity. References: Amulet IDE [24], Kleinecke 2019 [23], Kretzler 2022 [27], Saad 2021 [26], SWISS APERO [25], Mansour [28], Cheung 2019 [33], LAARGE 2020 [34]

**Figure 15:**
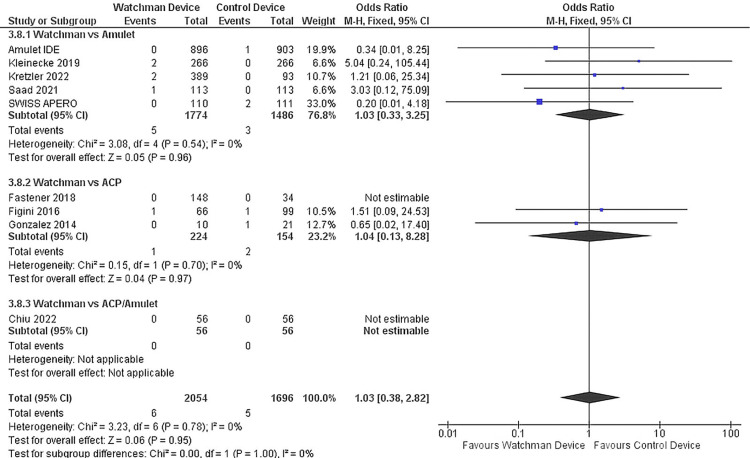
Procedure-related stroke; forest plot comparing procedure-related stroke incidence between Watchman and control devices; Rare events References: Amulet IDE [24], Kleinecke 2019 [23], Kretzler 2022 [27], Saad 2021 [26], SWISS APERO [25], Fastner 2018 [32], Figini 2016 [31], Gonzalez 2014 [29], Chiu 2022 [35]

**Figure 16:**
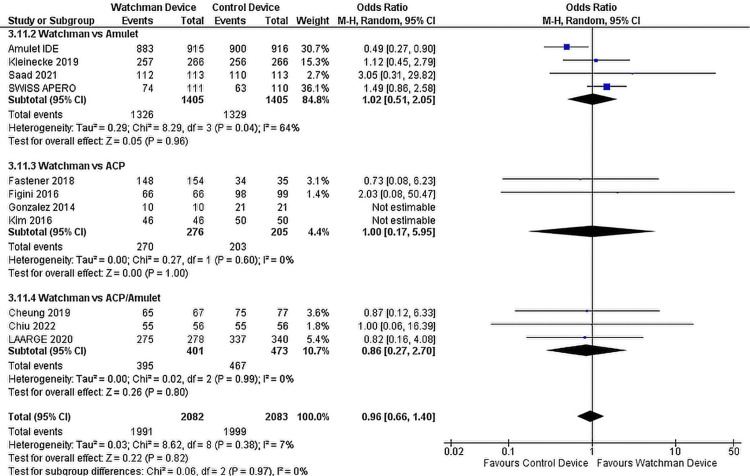
Procedural success; Forest plot comparing minor procedural success between Watchman and control devices; equivalent across devices References: Amulet IDE [24], Kleinecke 2019 [23], Saad 2021 [26], SWISS APERO [25], Fastner 2018 [32], Figini 2016 [31], Gonzalez 2014 [29], Cheung 2019 [33], Chiu 2022 [35], LAARGE 2020 [34].

**Figure 17:**
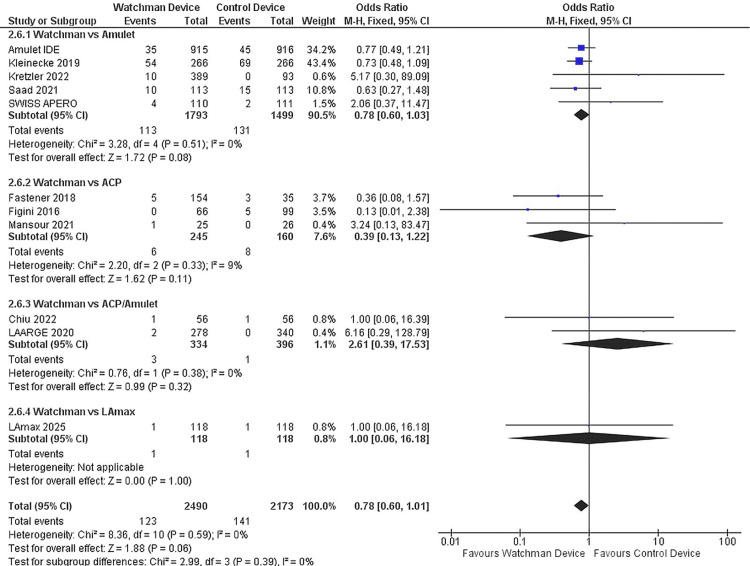
All-cause mortality device comparison; Forest plot comparing all-cause mortality incidence between Watchman and control devices; Borderline significant (p=0.06) References: Amulet IDE [24], Kleinecke 2019 [23], Kretzler 2022 [27], Saad 2021 [26], SWISS APERO [25], Fastner [32], Figini 2016 [31], Mansour [28], Chiu [35], LAARGE 2020 [34], LAmax [36].

## Appendices

Supplementary material 1

Supplementary material 2 

Supplementary material 3  

Supplementary material 4  

Supplementary material 5  

Supplementary material 6  

Supplementary material 7  

Supplementary material 8  

Supplementary material 9  
